# Re-evaluating the Need of Postoperative Blood Testing in Low-Risk Patients After Primary Elective Arthroplasty: A Single-Centre Retrospective Analysis

**DOI:** 10.7759/cureus.76364

**Published:** 2024-12-25

**Authors:** Reuben C Johnson, Inderpaul Samra, Nischay Keshava, Amit K Yadav, Janam Merchant, Vivek Thakker, Sunil Panchani

**Affiliations:** 1 Trauma and Orthopaedics, Wrightington Hospital, Wigan, GBR

**Keywords:** arthroplasty, blood tests, elective surgery, hip replacement, knee replacement, transfusion

## Abstract

Introduction

Increasing demand and financial burdens are placing significant strain on current health resources. To help ease pressures, there has been increased emphasis on improving patient flow and saving costs within the health service. Routine postoperative blood tests in otherwise healthy patients may add to delays and healthcare costs without influencing subsequent management. Recent studies suggest that routine postoperative blood tests may be unnecessary in fit and healthy patients undergoing elective arthroplasty. We aimed to assess this practice at our institution in the American Society of Anaesthesiologists (ASA) grade 1 and 2 patients undergoing elective hip and knee arthroplasty.

Methods

We conducted a retrospective review of 1595 consecutive elective hip and knee replacements in ASA 1 and ASA 2 patients at our institution over a one-year period from 2021 to 2022. Operation notes and electronic databases were analyzed to collect data regarding demographics, co-morbidities, treatment, pre and postoperative blood tests, and any documented interventions in these patients. Binomial logistic regression was employed to identify risk factors associated with postoperative abnormalities and the need for clinical intervention.

Results

Postoperative blood abnormalities were identified in 75.4% of patients, primarily anaemia (69.2%) and hyponatremia (29.9%). Anaemia was similarly prevalent in both total hip arthroplasty (THA) and total knee arthroplasty (TKA) patients, with 70.2% affected in each group, although the majority of cases were mild (83.8% in THA and 90.5% in TKA). Hyponatremia was significantly more common in TKA patients (40.3%) compared to THA patients (19.3%), although most cases were mild in severity. Only 5% of cases required any intervention, with higher rates observed in the TKA group compared to the THA group (p=0.008). Blood transfusion rates were low, occurring in 0.6% of cases. Factors associated with postoperative anaemia included higher BMI, preoperative antiplatelet use, and lower preoperative haemoglobin levels, while postoperative hyponatremia was linked to preoperative sodium levels, loop diuretic use, and PPI use. Acute Kidney Injury (AKI) was identified in 2.2% of patients and was predominantly mild. Potassium abnormalities were infrequent, with hypokalemia occurring in 1.5% of patients and no cases of hyperkalemia in our series.

Conclusions

Although postoperative blood test abnormalities were common, the majority were mild and rarely influenced management in this low-risk cohort of patients, with overall low postoperative intervention rates. Selective blood testing may allow safe targeted testing in this low-risk cohort of patients, minimizing costs and saving valuable resources.

## Introduction

The growing demand for elective joint arthroplasty, combined with increasing pressures on hospital bed availability, has placed a significant financial strain on healthcare systems [1]. As a result, there has been a strong focus on optimizing patient flow and reducing the length of hospital stays to alleviate these pressures [2]. One common practice within many enhanced recovery pathways following arthroplasty is the routine use of postoperative blood tests. These tests have become standard practice in assessing patients' recovery and detecting complications early, ensuring that potential issues are identified and treated promptly.

However, recent evidence has started to question the necessity of routine blood tests in the postoperative management of elective joint arthroplasty patients [3]. Various studies have examined the outcomes and complications associated with total hip arthroplasty (THA), total knee arthroplasty (TKA), and unicompartmental knee arthroplasty (UKA) procedures. These studies provide valuable insights into the rates of complications such as transfusions, electrolyte imbalances, and acute kidney injuries, as well as the necessity for intervention following abnormal test results. Studies suggest that these tests may not always be required and rarely impact clinical decision-making or management [4-7]. Despite this, many recovery protocols continue to include routine post-operative blood tests as part of standard care, often out of tradition rather than based on clinical necessity. As healthcare systems strive for greater efficiency and cost-effectiveness, the challenge lies in balancing the need for thorough patient monitoring without the potential overuse of resources. Eliminating unnecessary testing could not only reduce healthcare costs but also streamline patient care, shortening hospital stays and freeing up valuable resources.

The goals of this study were to determine the risk variables influencing postoperative blood results and to evaluate the necessity of routine post-op blood tests in American Society of Anaesthesiologists (ASA) grade 1 and 2 patients undergoing primary hip and knee arthroplasty.

## Materials and methods

This was a retrospective cohort comparison study aiming to evaluate the need for routine postoperative blood testing in American Society of Anaesthesiologists (ASA) grade 1 and 2 patients undergoing hip and knee arthroplasty at Wrightington Hospital in 2022. Patients with ASA classifications higher than 2, those undergoing revision surgeries, or those with incomplete medical records were excluded from the analysis.

Data was gathered retrospectively from electronic hospital records and operative and case notes. Details of preoperative and postoperative day 1 blood test results were recorded. The blood parameters considered included haemoglobin levels, electrolytes (sodium, potassium, chloride, magnesium), renal function tests (urea and creatinine, estimated glomerular filtration rate, (eGFR)), and coagulation profile (prothrombin time (PT), activated partial thromboplastin Time (APTT), international normalized ratio (INR)). The results were compared to identify any postoperative abnormalities or significant deviations from baseline. We collected information regarding patient comorbidities and regular medications to assess the association of variables with derangements in post-operative blood test results. Anaemia was graded as mild, moderate, and severe according to World Health Organisation (WHO) grading (<120 g/L for women and <130 g/L for men) [8]. Hyponatremia was graded as mild, moderate, and severe as per National Institute for Health and Care Excellence (NICE) definitions (Mild: serum sodium concentration of 130-135 mmol/L, Moderate: 125-129 mmol/L, Severe: serum sodium concentration of less than 125 mmol/L) [9]. Intraoperative data regarding the type of anaesthesia, use of tourniquet (for TKA), intraoperative use of tranexamic acid, and duration of surgery were also collected to determine associations with blood results.

The primary outcome of the study was the incidence of postoperative blood test abnormalities and intervention rates in this population. All interventions performed as a result of abnormal blood results were recorded and presented in this study. This included treatments such as oral or injectable iron for managing mild to moderate anaemia, and blood transfusion for severe anaemia. Management strategies also encompassed fluid restriction for mild hyponatremia, oral or intravenous correction of moderate to severe hyponatremia, hypokalemia, or hyperkalemia, as well as intravenous fluid therapy for acute kidney injury (AKI).

Statistical analysis

Data was analyzed using a statistical package for the social sciences (SPSS, version 22.0, IBM software). Shapiro-Wilk normality tests were conducted to assess data distribution. Binomial logistic regression analysis was performed to assess the effects of risk factors on primary outcome measures, including the occurrence of postoperative blood test abnormalities and interventions. Independent samples T-tests and Chi-Squared tests were employed to determine differences between nominal variable groups in subgroup analysis. A two-tailed 5% significance level was applied to assess for statistical significance.

## Results

A total of 1595 consecutive patients undergoing primary hip and knee arthroplasty during 2022 were included for analysis. As per our inclusion criteria, all patients were classified as ASA 1 and ASA 2. Our sample comprised 790 total hip arthroplasties (THA) and 805 total knee arthroplasties (TKA), as shown in Table 1. It describes the demographic and clinical data for patients included in our study. The mean age of THA was 64.8±8.5 years, and the mean age of TKA was 67.9±12.5 years. Of the 790 cases undergoing THA, 305 were male and 485 were female, whilst for the 805 cases undergoing TKA, 376 were male and 429 were female. The majority of patients in both groups were ASA 2 (88% for THA and 91% for TKA), with a smaller proportion of ASA 1 (12% for THA and 9% for TKA). The mean BMI for TKA patients was higher (30.9±10.3) than in THA patients (28.5±9.2). THA had a longer mean surgical time of 87.6±28 mins compared to TKA, 75.1±23.5 mins. Patients undergoing THA had a longer mean hospital stay of 3.8±1.8 days compared to TKA patients of 3.4±3.6 days. 

**Table 1:** Demographic and clinical details of included THA and TKA patients in this study. * significant at the 5% level, Independent samples T-Test used for continuous variables and Chi-squared test for categorical variables. THA: total hip arthroplasty; TKA: total knee arthroplasty.

Variable	THA (n=790)	TKA (n=805)	p-value
Age (mean±SD)	64.8 ± 8.5	67.9 ±12.5	<0.0001*
Males (n, %)	305 (39%)	376 (47%)	<0.0001*
Females (n, %)	485 (61%)	429 (53%)	
ASA 1 (n, %)	95 (12%)	69 (9%)	0.023*
ASA 2 (n, %)	695 (88%)	736 (91%)	
BMI (mean±SD)	28.5±9.2	30.9±10.3	<0.0001*
Surgical time (mins) (mean±SD)	87.6±28.0	75.1±23.5	<0.0001*
Length of stay (days) (mean±SD)	3.8±1.8	3.4±3.6	<0.001*

**Figure 1:**
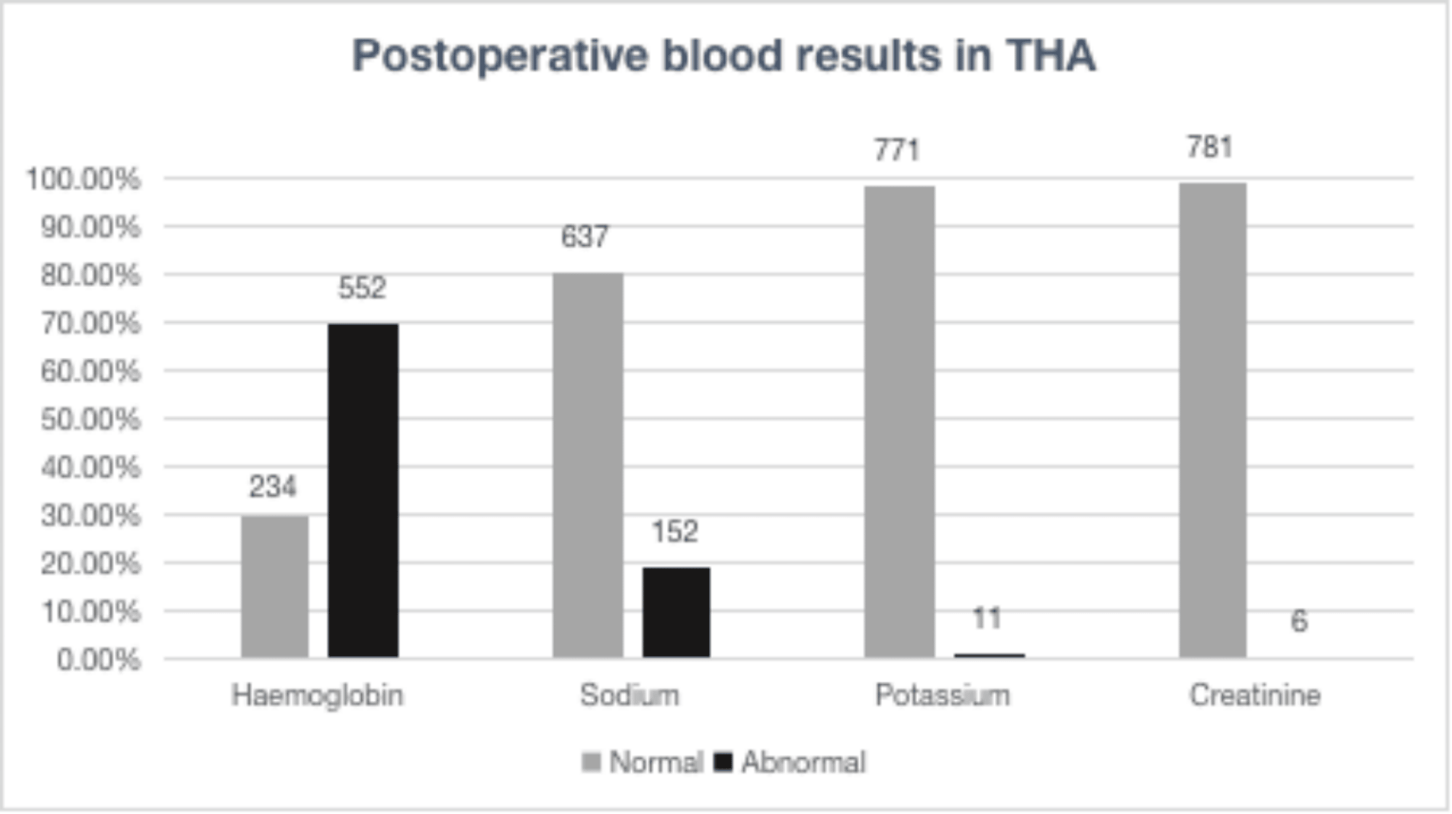
Postoperative blood derangements post THA (n=790) THA: total hip arthroplasty.

Overall, 75.4% (1203/1595) cases had abnormalities in postoperative blood tests outside of the normal limits. The majority of these derangements were mild in severity. Figure 1 shows postoperative blood results in THA.

**Figure 2:**
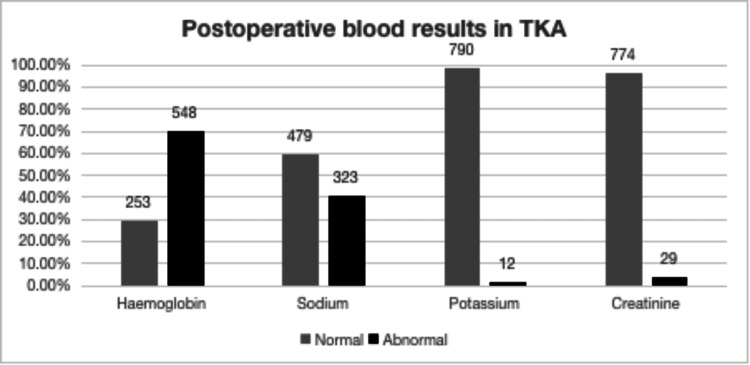
Postoperative blood derangements post TKA (n=805) TKA: total knee arthroplasty.

Figure 2 shows postoperative blood results in TKA.

Postoperative day 1 blood results for both THA and TKA patients were analysed and compared, focusing on blood abnormalities that most frequently required intervention. The primary issues identified were low haemoglobin levels and electrolyte imbalances. The figures above display the percentage of abnormal blood results in both groups.

In the THA group, 70.2% of patients (552/786) showed anaemia, 19.3% had hyponatremia (152/789), 1.4% exhibited hypokalemia (11/782) and 0.76% AKI (6/787). In contrast, the TKA group presented similar rates of anaemia at 70.2% (235/783, p>0.05) but higher rates of electrolyte and renal abnormalities with hyponatremia at 40.3%, (323/802) p<0.0001 and AKI at 3.6%, (29/803) p<0.0001. This also translated into increased intervention rates in patients undergoing TKA p=0.008). 

Table 2 shows the average postoperative haemoglobin decrease for THA and TKA patients. In THA, the mean haemoglobin drop was 19.6±12.7 mg/dL, while in TKA, it was slightly lower at 17.9±8.9 mg/dL, indicating a marginally greater haemoglobin reduction in THA patients compared to TKA. 

**Table 2:** Mean Haemoglobin drop in THA and TKA cases. *significant at the 5% level, Independent Samples T-Test used to assess statistical significance. THA: total hip arthroplasty; TKA: total knee arthroplasty.

	Preoperative Hb (g/l) (mean±SD)	Postoperative Hb (g/l) (mean±SD)	Hb Change (g/l) (mean±SD)	p-value
THA (n=790)	137.1±12.4	117.7±12.7	-19.6±12.7	<0.0001*
TKA (n=805)	135.6±11.1	117.4±15.6	-17.9±8.9	<0.0001*

Overall, hyponatremia occurred in 29.9% (475/1591) of patients. There was a higher incidence in the TKA group, 40.3% (323/802), compared with 19.3% (152/789) in the THA group (p <0.0001). The grades of hyponatremia observed in postoperative blood results for both THA and TKA patients are described in Table 3. Overall, mild hyponatremia was the most common grade in both THA and TKA groups. The TKA patients had a higher incidence of mild hyponatremia (92.9% vs 88.8%) than in the THA group. Conversely, moderate hyponatremia was more common in the THA group (8.6% vs. 4.6%) than in the TKA group, while the incidence of severe hyponatremia was comparable between the two groups (2.6% vs. 2.5%).

**Table 3:** Grading of hyponatremia in post-operative blood tests. *significant at the 5% level, Chi-squared test to assess for significance between groups. THA: total hip arthroplasty, TKA: total knee arthroplasty.

Hyponatremia grade [mmol/L]	THA (n=152)	TKA (n=323)	p value
Mild (130-134)	135 (88.8)	300 (92.9)	<0.0001*
Moderate (125-129)	13 (8.6)	15 (4.6)	0.74
Severe (<125)	4 (2.6)	8 (2.5)	0.26

Post-operative anaemia occurred in 69.2% of patients post-operatively (1098/1587). The incidence of anaemia was similar between THA and TKA groups, with no significant differences (p=0.45). Most cases were mild, with 83.8% (462/551) in the THA group and 90.5% (495/547) in the TKA group. Only three patients in total had severe post-operative anaemia, with 0.4% (2/551) in the THA group and 0.2% (1/547) in the TKA group (Table 4). 

**Table 4:** Grading of anaemia in post-operative blood tests. *significant at the 5% level, Chi-squared test to assess for significance between groups. THA: total hip arthroplasty; TKA: total knee arthroplasty.

Anaemia grade	THA (551)	TKA (547)	p-value
Mild (100-130 g/L)	462 (83.8)	495 (90.5)	0.22
Moderate (80-100 g/L)	87 (15.8)	51 (9.3)	0.009*
Severe (<80 g/L)	2 (0.4)	1 (0.2)	0.55

Overall, AKI incidence was low in this series, with 2.2% (35/1590) experiencing AKI (>1.5x rise in baseline serum creatinine). Only 1/1590 patients had a grade 2 AKI (2-2.5x rise in baseline creatinine). This patient had a raised BMI>35, underwent a TKA with a tourniquet, and had no other major co-morbidities. Rates of potassium abnormalities were also low, with postoperative hypokalemia found in 1.5% of patients overall (23/1584). Of these, 19 patients had mild hypokalemia (3-3.5 mmol/L) and 4 patients had moderate hypokalemia (2.5-2.9 mmol/L). No patients had severe hypokalemia, and no cases of hyperkalemia were identified postoperatively in our cohort.

Risk factors for blood abnormalities

Table 5 demonstrates factors associated with post-operative anaemia through binomial logistic regression. We identified several factors that were associated with anaemia. Each unit increase in BMI was associated with a 6% increase in the odds of anaemia occurring post-operatively. Interestingly, a decrease in surgical time was associated with a 1% increase in the odds of postoperative anaemia. The use of preoperative antiplatelet medications such as clopidogrel and aspirin was associated with a significant increase in postoperative anemia. Preoperative haemoglobin count was also associated with an increased likelihood of postoperative anaemia. 

**Table 5:** Adjusted odds ratios of risk factors associated with post-operative anaemia through binomial logistic regression. * significant at the 5% level. NSAIDs: nonsteroidal anti-inflammatory drugs.

Factor	Adjusted odds ratio (95% CI)	p-value
Age	1.00 (0.99-1.01)	0.704
BMI	1.06 (1.03-1.08)	<0.0001*
Surgical time	0.99 (0.98-1.00)	0.002*
Preoperative clopidogrel	3.87 (1.14-13.16)	0.027*
Preoperative aspirin	2.38 (1.21-4.71)	0.011*
Preoperative DOAC	0.58 (0.30-1.13)	0.10
Preoperative warfarin	2.15 (0.61-7.58)	0.24
NSAID use	1.66 (0.57-4.85)	0.35
Renal disease	0.78 (0.18-3.33)	0.73
Chronic inflammatory arthritis	0.65 (0.20-2.05)	0.46
Preoperative haemoglobin	1.11 (1.09-1.12)	<0.0001*
Preoperative platelet count	1.00 (0.99-1.00)	0.24

On subgroup analysis, categorical risk factors identified for moderate/severe anaemia (Hb<100 g/L) included the use of NSAIDs (χ^2^=4.76, p=0.029), THA surgery (χ^2^=11.44, p=0.001), Surgical time >90 mins (χ^2^=11.44, p<0.0001), and preoperative anaemia (χ^2^=58.00, p<0.0001). Patients with a postoperative moderate/severe anaemia overall had lower BMI (27.9 vs 30.0, p=0.017), longer surgical time (90.1 vs 80.7 mins, p<0.0001), and lower preoperative Hb (124.5 vs 137.5, p<0.0001).

Table 6demonstrates risk factors for post-operative hyponatremia. We identified several factors that were associated with the risk of hyponatremia postoperatively. Patients taking loop diuretics and/or proton pump Inhibitors (PPI) had increased risks of developing post-operative hyponatremia. Preoperative sodium was associated with post-operative hyponatremia. Interestingly, age was associated with a mild reduction in the risk of postoperative hyponatremia in our logistic regression model.

**Table 6:** Adjusted odds ratios of risk factors associated with post-operative hyponatremia through binomial logistic regression. * significant at the 5% level. BMI: body mass index, ACEi: angiotensin-converting enzyme inhibitor, NSAIDs: nonsteroidal anti-inflammatory drugs.

Factor	Adjusted odds ratio (95% CI)	p-value
Age	0.98 (0.96-0.99)	<0.0001*
BMI	1.00 (0.98-1.01)	0.42
Surgical time	1.00 (0.99-1.01)	0.31
Renal disease	0.51 (0.14-1.93)	0.32
Hypothyroidism	1.01 (0.64-1.58)	0.97
NSAID use	2.80 (0.77-10.18)	0.12
PPI use	1.66 (1.19-2.31)	0.003*
Anti-depressant use	1.89 (0.81-4.42)	0.14
ACEi use	1.08 (0.78-1.50)	0.66
Loop diuretic use	8.28 (1.05-65.3)	0.045*
Thiazide diuretic use	0.94 (0.57-1.56)	0.81
Preoperative Sodium	1.25 (1.19-1.30)	<0.0001*

Subgroup analysis of cases with moderate/severe hyponatremia revealed patients with pre-existing renal disease (χ^2^=9.88, p=0.002) and preoperative hyponatremia (χ^2 ^=113.33, p<0.0001) were at significant risk. Overall, patients with moderate/severe hyponatremia had reduced BMI (26.8 vs 27.8, p <0.0001), Increased age (71.2 vs 66.3, p<0.0001), and lower preoperative sodium levels (135.7 vs 139.3 p<0.0001).

Interventions

A total of 5% of cases required interventions for blood test abnormalities post-operatively out of the total cohort (80/1595), with a difference between groups with 52 required in TKAs and 28 in THA patients (chi-squared, p-0.008). Out of the 80 patients requiring interventions, 2.5% were ASA 1 (2/80), and 97.5% (78/80) were ASA 2 (χ^2^=5.56, p=0.018). A breakdown of the combinations of interventions required post-operatively is given in Table 7. Incidental medical complications included 10 patients with postoperative pneumonia, six cardiac arrhythmias, three urinary tract infections, and three symptomatic venous thromboembolisms. We had no intrahospital mortality in this cohort. We found no association between recorded risk factors and the development of postoperative medical complications. On subgroup analysis, only increased age (69.0±11.4 vs 66.3±10.8, p=0.029) and lower preoperative eGFR (78.1±14.0 vs 82.0±10.9, p=0.025) were factors associated with intervention requirement. 0.6% of patients (10/1595) required postoperative transfusion in our series, with no significant difference between THA and TKA groups. We found no significant factors affecting transfusion risk in our study with the small number of cases involved.

**Table 7:** Interventions required in THA and TKA patients. THA: total hip arthroplasty; TKA: total knee arthroplasty.

Intervention (n=80)	THA Group ( n=790)	TKA Group ( n=805)
Blood transfusion	6	4
IV iron	2	1
Oral iron	1	1
Oral sodium supplementation	1	3
Fluid restriction	12	30
IV fluids	2	8
Oral potassium replacement	4	3
Oral magnesium replacement	3	2

Table 8 demonstrates the analysis of factors associated with required postoperative interventions and reveals several significant findings. BMI was found to have a small yet significant impact, with each unit increase associated with a 2% reduction in the odds of intervention (OR 0.98, 95% CI 0.97-1.00, p=0.010). ACE inhibitor use was strongly associated with increased odds of intervention, with an odds ratio of 2.42 (95% CI 1.35-4.33, p = 0.003). Preoperative sodium levels were also identified as a significant predictor, with higher levels associated with an increased risk of intervention (OR 1.10, 95% CI 1.01-1.19, p=0.021). Although age (OR 0.98, 95% CI 0.95-1.00, p=0.053) and renal disease (OR 4.67, 95% CI 0.90-24.19, p=0.066) showed trends towards statistical significance, neither reached the threshold for formal significance. Other factors, including surgical time, cardiovascular disease, diabetes, and the use of various preoperative medications such as aspirin, clopidogrel, and warfarin, did not demonstrate a significant association with postoperative intervention requirements. Similarly, variables like preoperative potassium, urea, creatinine, and haemoglobin levels showed no predictive value in this cohort. This highlights the multifactorial nature of interventions, with some risk factors warranting closer clinical attention, particularly preoperative sodium levels and the use of ACE inhibitors.

**Table 8:** Adjusted odds ratios of risk factors associated with post-operative interventions through binomial logistic regression. * significant at the 5% level. BMI: body mass index, ACEi: angiotensin-converting enzyme inhibitor, PPI: proton pump inhibitor.

Factor	Adjusted odds ratio (95% C.I.)	p-value
Age	0.98 (0.95-1.00)	0.053
BMI	0.98 (0.97-1.00)	0.010*
Surgical time	1.00 (0.99-1.00)	0.26
Renal disease	4.67 (0.90-24.19)	0.066
Cardiovascular disease	1.55 (0.52-4.60)	0.43
Diabetes	1.31 (0.73-4.61)	0.67
PPI use	0.94 (0.50-1.75)	0.84
ACEi use	2.42 (1.35-4.33)	0.003*
Loop diuretic use	0.66 (0.07-5.54)	0.70
Thiazide diuretic use	1.88 (0.85-4.17)	0.12
Preoperative aspirin	1.05 (0.35-3.18)	0.93
Preoperative clopidogrel	1.64 (0.35-7.77)	0.53
Preoperative direct oral anticoagulants (DOAC)	1.30 (0.33-5.06)	0.71
Preoperative warfarin	2.59 (0.49-13.55)	0.26
Calcium channel blocker use	1.12 (0.61-2.07)	0.71
ß blocker use	0.36 (0.11-1.14)	0.082
Preoperative haemoglobin	1.00 (0.98-1.03)	0.70
Preoperative sodium	1.10 (1.01-1.19)	0.021*
Preoperative potassium	1.12 (0.61-2.05)	0.71
Preoperative urea	1.12 (0.93-1.35)	0.24
Preoperative creatinine	1.00 (0.98-1.02)	0.91

## Discussion

The National Health Service (NHS) currently faces significant financial pressures and constraints due to increasing demands and limitations in available funding. A review of NHS pathology services in England identified major inefficiencies and made several recommendations to improve service quality, cost-effectiveness, and patient outcomes [10]. Evidence suggests that up to 40% of blood tests may be unnecessary [11]. Reducing unnecessary tests, therefore, without compromising patient care, could lead to substantial cost savings and more efficient use of resources. 

This study aimed to evaluate the necessity of routine postoperative blood tests in patients undergoing primary total hip arthroplasty (THA) and total knee arthroplasty (TKA). Our findings suggest that while postoperative abnormalities in blood parameters are common, they are frequently mild and rarely necessitate intervention in our cohort of patients. These results prompt a re-evaluation of the routine use of blood tests in this patient population, particularly in light of the associated resource use and cost implications. A key finding of our analysis was that 75.4% of patients had at least one abnormal blood result postoperatively, with anaemia and hyponatremia being the most frequently observed abnormalities, yet our intervention rate overall was only 5%, with the majority of interventions for treatment of mild blood derangements. Only 0.19% (3/1584) had severe post-operative anaemia (Hb <80 g/L), 0.75% had severe hyponatremia (12/1591), and 2.2% (35/1590) with a mild AKI. This is consistent with multiple reports which demonstrate that blood test abnormalities are frequent but rarely require intervention [3-7,12-13]. 

Postoperative anaemia, as defined by the WHO occurred in nearly 70% of cases, but the vast majority were mild (THA 83.8% vs TKA 90.5%), with only three patients experiencing severe anaemia requiring blood transfusion. Moreover, the haemoglobin drop was comparable between THA and TKA, with a mean decrease of approximately 18-20 g/dL. Despite the frequency of anaemia, the clinical impact was minimal, with only 0.6% of patients requiring transfusion. Perioperative anaemia has been linked to inferior outcomes and increased complication risk [14-15].

Our blood transfusion rates of 0.6% overall concur with existing literature [3-4,12,16]. Point of care testing (POCT), which can be performed in recovery, is one potential means through which routine laboratory testing may be avoided, and patients who may benefit from a transfusion can be identified [17]. Some centres have successfully included POCT as part of their day case surgery protocols, however the specificity of identifying patients with low haemoglobin can be variable between devices [18] and some meta-analyses suggest exercising caution when utilising these instead of standardised testing [19]. Risk factors for postoperative anaemia included higher BMI, preoperative antiplatelet use, and lower preoperative haemoglobin levels. Raised BMI leading to post-operative anaemia may be partly explained by the potential need of larger incisions and some studies have found BMI to lead to increased bleeding risk [20]. Although antiplatelet agents are stopped prior to surgery to mitigate bleeding risk, our study demonstrates reduced post-operative haemoglobin in these patients. Recovery of platelet function requires in vivo replacement of impaired platelets that have been irreversibly affected by these agents. We found lower preoperative haemoglobin levels were associated with anaemia post-operatively and highly associated with moderate to severe anaemia cases. Although we did not find a direct link between preoperative anaemia and the occurrence of post-operative complications (which could be due to the low numbers of complications post-surgery), we found that preoperative haemoglobin levels were negatively correlated with LOS. Several studies have highlighted the importance of correcting preoperative anaemia due to perioperative morbidity and mortality risk in addition to LOS [14,21]. Interestingly, surgical time was not identified on logistic regression as a risk factor for anaemia which may be due to universal perioperative tranexamic acid administration, which is known to reduce blood loss [22]. Also, this could be due to emphasis on intraoperative haemostasis during surgery leading to longer surgical time. We did not account for amount of intraoperative blood loss in this series due to its retrospective nature. 

Hyponatremia was another common finding, particularly in the TKA cohort, where it affected 40.3% of patients compared to 19.3% in the THA group. The majority of cases were mild, however research has shown that even normal or low-normal serum sodium levels (e.g., 133 to 137 mmol/L) can be associated with longer hospital admissions, along with marked increases in perioperative morbidity and mortality [23-24].

A higher incidence of hyponatremia may be reflective of increased stress and pain levels in TKA patients postoperatively, which can impact ADH levels and lead to sodium imbalance [25-26]. The higher incidence could also be explained by the significant difference in ASA grades between THA and TKA groups, with more ASA 2 patients in the TKA group and a higher incidence of renal disease (p-0.006). Variations in IV fluid administration perioperatively may also account for the differences between groups. Although most cases were mild, moderate hyponatremia was slightly more prevalent in THA patients, while severe hyponatremia was extremely rare in both groups. Factors such as preoperative sodium levels, loop diuretic use, and proton pump inhibitor use were associated with an increased risk of hyponatremia, as confirmed by logistic regression analysis. Although more commonly associated with electrolyte imbalances, thiazide diuretics had no statistically significant effect on our results. The occurrence of hyponatremia translated to a relatively low rate of interventions overall, with only 2.6% of patients requiring measures such as fluid restriction or sodium supplementation. Some authors have suggested that cases of mild hyponatremia can be safely managed in the community without risk of complication and/or readmission [27].

Renal complications, including AKI, were infrequent, with an incidence of 2.2%. Most cases of AKI were mild and resolved without long-term sequelae, underscoring the low overall risk in this cohort of ASA 1 and 2 patients. The incidence of AKI was significantly higher in the TKA group, echoing findings from Lara et al. [28]. Similarly, potassium abnormalities were rare, with mild hypokalemia observed in only 1.5% of patients and no instances of severe hypokalemia. These findings echo other large series showing similar findings [3-4,12]. 

Overall, the need for postoperative interventions was low, with only 5% of patients requiring any treatment. Interventions were more common in TKA patients, driven by higher rates of electrolyte abnormalities. Logistic regression identified increased preoperative sodium levels, ACE inhibitor use, and lower BMI as predictors of intervention, highlighting specific patient groups that may benefit from targeted postoperative monitoring.

Some authors have attempted to produce predictive scoring systems for selective postoperative testing [29-30]. Both systems have been intended to identify patients at risk of transfusion with success utilizing author datasets. Wu et al. [29] demonstrated 86.95% sensitivity and 40% specificity in their series, whilst the total hip arthroplasty blood test usefulness score (THABUS) system demonstrated a sensitivity of 96.65% and specificity of 75.54% [30]. We attempted to apply these models to our dataset. However, we do not routinely perform liver function tests to apply the full Wu et al. scoring system, and we did not record osteonecrosis as an indication for THA in our series. However, modifying these scores to consider all remaining parameters did not identify transfusion patients in our dataset. Ultimately this is likely due to our very low number of severe anaemias and missing data required for the full scores as given by the authors, however, it could also be the generalisability of these scoring systems may not be applicable to all datasets. We suggest further work should be performed, possibly using AI machine learning to better predict patients who could benefit from selected testing.

Limitations

Our study has several limitations. Firstly, our study was a single-centre retrospective study, which leaves the study open to bias and may limit the generalisability of our findings. Being retrospective in nature, accurate assessments of intraoperative blood loss were not possible. However, we had low levels of missing data overall in our dataset. We did not look at indications of THA and did not collect information regarding liver function tests, meaning that we could not fully apply some existing predictive scoring systems to our data. However, our results are a real-life pragmatic snapshot of preoperative and postoperative blood tests available within our healthcare system. Overall, we had low transfusion rates, meaning we were not able to find risk factors that led to increased transfusion risk. We only included ASA 1 and 2 patients in our series as we felt that higher-risk ASA 3 and 4 patients should be tested postoperatively given their lower physiological reserve. Until robust and validated scoring systems exist which minimize false negatives, the risks of missing blood test abnormalities in this higher-risk cohort outweigh the cost benefits. 

## Conclusions

Whilst postoperative blood test abnormalities are common in THA and TKA patients, the vast majority are mild and do not require any further interventions in ASA 1 and ASA 2 patients. A selective approach to postoperative blood testing in these patients could be adopted, focusing on higher-risk individuals, and is likely to be more cost-effective and efficient while maintaining patient safety.

Our findings have important implications for clinical practice. Routine testing in all arthroplasty patients appears to yield limited actionable information, particularly in patients without identifiable risk factors. This study supports a more selective approach, reserving routine tests for older patients, those with raised BMI, preoperative anaemia and hyponatremia, patients with preexisting renal disease, or those on medications associated with increased risk of blood abnormalities, such as ACE inhibitors, diuretics, and antiplatelet agents. A targeted approach could lead to considerable cost savings, streamline patient care, shorten hospital stays, and optimize resources. Further research is warranted to validate these findings and refine guidelines for postoperative monitoring in arthroplasty patients.
